# Quantitative Analysis and Comparison of BMI among Han, Tibetan, and Uygur University Students in Northwest China

**DOI:** 10.1155/2013/180863

**Published:** 2013-12-17

**Authors:** Bai Jingya, He Ye, Wang Jing, Huanjiu Xi, Hai Tao

**Affiliations:** ^1^Faulty of Medicine, Northwest University for Nationalities, China; ^2^Centre for Modern Languages and Human Science, University Malaysia Pahang, Malaysia; ^3^College of Anatomy, Liaoning Medical University, China

## Abstract

*Objectives*. To fully analyze and compare BMI among Han, Tibetan, and Uygur university students, to discuss the differences in their physical properties and physical health, and thus to provide some theoretical suggestions for the improvement of students' physical health. *Methods*. The cross-sectional random cluster sampling was used to investigate 10103 Han, Tibetan, and Uygur university students, aged 20–24 in Northwest China, and their height and weight were measured to calculate BMI. The BMI classification criteria for Chinese established by Work Group on Obesity in China (WGOC) were used for screening. *Results*. Han, Tibetan, and Uygur university students show low obesity rates but high overweight rates. Han, Tibetan, and Uygur university students present a high rate of underweight, normal weight, and overweight, respectively. Female Han students show higher underweight and normal weight rates, but lower overweight and obesity rates, than male Han students. Female Tibetan students show higher normal weight rate, but lower overweight and obesity rates, than male Tibetan students. BMI increases with age for male students but decreases with age for female students. Male Uygur students show higher obesity rate than female Uygur students. Tibetan and Uygur university students have higher BMI than other minorities in South China.

## 1. Introduction

As the final stage in puberty, universities are the key period for the development of the physical and psychological health as well as the physical quality. The growth and development levels and the health conditions in this stage significantly impact on physical quality, psychological quality, employment and career selection, and daily life and production [1]. Recently, the investigation and research of the physical health of university students throughout China show that their physical health continue to decline, which is inconsistent with the demands of rapid economic development and quality education. Since university students are backbones of China, their physical health relates to not only themselves but also the nations and the country. Global practices and researches indicate that it is effective to prevent health problems by improving physical level. Therefore, it is critical to explore the rules of body and mind development of university students, and the improvement of their physical health has become the key for state-level adolescent health, as well as the hot issues in physical studies.

BMI is an important index to evaluate levels of the bodies' morphological development, nutritional status, and bodies' symmetry degree, which is closely related to fat contents, long-term health effects, and disease risks. BMI is superior for simply calculation, easy measurement, low costs, and good reproducibility. At the same time, it shows good resolution among different ages, genders, races, nation, and development and maturity levels. Therefore, BMI can be used as a predictor for assessing obesity and relevant diseases in adulthood [2]. BMI is globally applied as a screening index and is widely applied in population screening and large-scale epidemiological surveys [3].

Due to such acquired elements as environment, nutrition, hygiene, and physical exercises, university students from different areas and nations show distinct physical properties. To fully understand their physical conditions, especially the interregional and interethnic physical characteristics and laws, this paper will discuss causes for differences in physical health will provide some theoretical basis and suggestions for further improving their physical conditions and for sports education reforms in colleges and universities.

## 2. Subjects

Han, with a population of 1.3 billion, is the first nation in China and even in the world. Tibetan and Uygur are two important minorities in Northwest China and have unique languages, beliefs, festivals, and customs. Tibetans dwell throughout China but mainly in Tibet Autonomous Region. Among 5.41 million Tibetans, 1.35% has received junior college or college education. Among 8.39 million Uygurs, 2.74% has received junior college or college education. Uygurs reside throughout the country but mainly in the Xinjiang Autonomous Region. As the future and hope of a nation, university students undertake the mission for inheriting, delivering, criticizing, and innovating their own ethnic cultures. University education concerns not only the survival and development of a nation, but also the collaborative development and prosperity of all nations in China. Han, Tibetan, and Uygur university students were selected as the subjects of this paper.

Cross-sectional random cluster sampling was used to select university students aged 20–24 in Northwest China in September 2011. The subjects are all physically healthy and both parents are the same nation as the child. For instance, parents of a Han student are both Han. Moreover, Tibetan and Uygur university students are from and extended living in minority areas. Prior to the investigation, we got permission from the school directors of the students' affairs divisions, the overall arrangements from the school doctors, and agreement from the students and parents. Strict quality control measures were guaranteed for point selection, detection periods, testing methods, and types of instruments. Nation, gender, native place, date of birth, and grade were collected from each subject. After physical examination and medical history inquiry, subjects with chronic diseases in major organs, disability, or developmental obstacles were excluded. The valid sample number is 10,103, with 5,328 males and 4,775 females, aged 22.56 ± 1.41. The subjects include 7,937 Han students (4,419 males and 3,518 females), 1,376 Tibetan students (642 males and 734 females), and 790 Uygur students (267 males and 523 females), as shown in Table 1.

## 3. Methods

Primary analysis will be via intention to treat with all participants included regardless of dropout or level of adherence. Missing data will be imputed according to the maximum likelihood expectation algorithm via the Statistical Package for the Social Sciences (IBM, SPSS version 19.0). Data will be presented as the mean ± standard deviation or median and range, as appropriate. Confidence intervals will be used to express group differences.

Measure of body shape indices: the height and weight of each subject was measured and recorded according to the methods in Human Body Measurement Manual by Shao [4]. Each index was measured three times to calculate an average. The specific methods and the meaning are as follows.

 
*Height* reflects the developmental level of human skeleton and the longitudinal developmental level of a mortal body. A sitting height meter was used. Each subject took off shoes and hat and stood at attention on the floor, with hands in natural fall, heels together, toes at 45°, heels, hips, and shoulders closely against the upright post, trunk naturally straight, and eyes looking at the front horizontally. As a subject stood on the right, move the slip test plate slowly until it touched the top of the head. Height was measured in centimeter, with an error less than 0.5 cm.
 
*Weight* reflects the transverse developmental level of the human body. A lever balance was used. Each subject relieved the bowels in advance, wore underwear, and stood on the platform barefoot. Hands should not touch anything. Adjust the weights until the lever balanced and record the reading to the minimum scale. Weight was measured in kilogram, with an error less than 0.1 kg.
 
*BMI screening*: BMI was calculated as BMI = weight (kg)/(height (m))^2^.

The dividing standards for overweight and obesity are quite different globally. BMI standards for overweight and obesity are 25 kg/m^2^ and 30 kg/m^2^, respectively, in Western countries [5], and 23 kg/m^2^ and 25 kg/m^2^, respectively, in Asian countries [6]. Based on surveys on 239,972 people aged 20–70 in 1990s covering 21 provinces, WGOC proposed BMI standards for Chinese adults [7]: BMI ≤ 18.5 is underweight, 18.5 ≤ BMI ≤ 24 is normal weight, BMI ≥ 24 is overweight, and BMI ≥ 28 is obesity. Therefore, the subjects were divided into underweight, normal weight, overweight, and obesity groups according to the BMI classification criteria by WGOC.

## 4. Results

The data were entered, arranged, checked, and calibrated by specially assigned persons and were then statistically analyzed. Excel was used for building database for input and SPSS 19.0 was used for statistical analysis. *t*-test was used for average comparison between two groups, and *χ* ^2^-test and Fisher's exact probability method were used for probability comparison. The widely used linear regression quantifies the interdependence between two or more variables by using mathematical statistics.

### 4.1. General Comparison of BMI Distributions of Han, Tibetan, and Uygur University Students

The university students of the three nations show differences in the rates of underweight (*χ* ^2^ = 7.46, *P* < 0.05), normal weight (*χ* ^2^ = 10.86, *P* < 0.01), and overweight (*χ* ^2^ = 8.90, *P* < 0.05). Underweight rate is higher in Han students than in Tibetan students (*χ* ^2^ = 7.30, *P* < 0.01). Normal weight rate is higher in Tibetan students than in Han students (*χ* ^2^ = 5.83, *P* < 0.05) and Uygur students (*χ* ^2^ = 10.50, *P* < 0.01). Overweight rate is higher in Uygur students than in Han students (*χ* ^2^ = 8.43, *P* < 0.01) and Tibetan students (*χ* ^2^ = 6.43, *P* < 0.05), as shown in Table 2.

### 4.2. Gender Comparison of BMI Distributions of Han, Tibetan, and Uygur University Students

Female Han, Tibetan, and Uygur students show differences in the rates of underweight (*χ* ^2^ = 7.13, *P* < 0.05), normal weight (*χ* ^2^ = 12.01, *P* < 0.01), and overweight (*χ* ^2^ = 14.15, *P* < 0.01). Malnutrition rate is higher in female Han students than in female Tibetan students (*χ* ^2^ = 7.03, *P* < 0.01). Normal weight rate is higher in female Tibetan students than in Han (*χ* ^2^ = 4.60, *P* < 0.05) and Uygur (*χ* ^2^ = 10.5, *P* < 0.01) female students. Normal weight rate is higher in female Han students than in female Uygur students (*χ* ^2^ = 5.60, *P* < 0.05). Overweight rate is higher in female Uygur students than in Han (*χ* ^2^ = 13.73, *P* < 0.01) and Tibetan (*χ* ^2^ = 7.83, *P* < 0.01) female students.

### 4.3. Intraethnic Gender Comparison of BMI Distributions

Malnutrition rate (*χ* ^2^ = 15.55, *P* < 0.01) and normal weight rate (*χ* ^2^ = 11.21, *P* < 0.01) are higher in female Han students than in male Han students. Overweight rate (*χ* ^2^ = 39.03, *P* < 0.01) and obesity rate (*χ* ^2^ = 37.16, *P* < 0.01) are higher in male Han students than in female Han students.

Normal weight rate is higher in female Tibetan students than in male Tibetan students (*χ* ^2^ = 5.4, *P* < 0.05). Overweight rate (*χ* ^2^ = 7.62, *P* < 0.05) and obesity rate (*χ* ^2^ = 6.57, *P* < 0.01) are higher in male Tibetan students than in female Tibetan students (Table 2).

Obesity rate is higher in male Uygur students than in female Uygur students (*χ* ^2^ = 4.62, *P* < 0.05) (Table 2).

### 4.4. BMI-Age Relationship

Han, Tibetan, and Uygur university students were grouped by gender for overall analysis. One-factor analysis of variance indicates significant BMI differences among different ages for either gender (Table 3). (male university students: *F* = 6.591, *P* = 0.000; female university students: *F* = 3.332, *P* = 0.010). Linear regression analysis indicates the relationships between BMI and age for either gender. Linear regression for male university students is *Y* = 18.280 + 0.142*X* (*F* = 23.774, *R* = 0.067, *P* = 0.0000); Linear regression for female university students is *Y* = 22.503 − 0.078*X* (*F* = 8.670, *R* = 0.043, *P* = 0.003).

### 4.5. Comparison of BMI Averages between Tibetan, Uygur University Students and Other Nations in South China

In Table 4, the comparison of BMI is used to Tibetan, Uygur, Ethnic minorities in Yunnan, Yi nationality and Sichuan Qiang. The results show Tibetan and Uygur university students have higher BMI than the minorities in South China both male and female.

### 4.6. Comparison among BMI, Gender and Age in Tibetan, Uygur and Han University Students

In Table 5, the average of BMI increases with age in the three nationalities' male university students, but female trends are running counter to male university students. The comparison results shows that the average values of BMI of Uygur male university students are higher than Tibetan in 20-year-old group (*P* = 0.029). The compared results demonstrates that the average values of BMI of Tibetan female university students are higher than Han in 20-year-old group (*P* = 0.034). The comparison results shows that the average values of BMI of Tibetan male university students are higher than Han in 24-year-old group (*P* = 0.039). In the total results, the average values of BMI of Uygur male university students are higher than Han (*P* = 0.007) and Tibetan (*P* = 0.021). The average values of BMI of Tibetan and Uygur female university students are higher than Han (*P* = 0.011).

## 5. Discussion

University students enjoy an independent lifestyle, but such freedom brings some unhealthy eating and behavioral habits, which will lead to overweight, obesity, or malnutrition [8–12]. Health harms of underweight and obesity include physical, psychological, and behavioral aspects, while underweight will increase risks for digestive system disease, low immunity, and anemia. Obesity is the major cause for many chronic noncommunicable diseases, such as hypertension, diabetes, hyperlipemia, cardiovascular and cerebrovascular diseases, and cancer [13, 14].

BMI is related to the two shape indices of height and weight. About 75% of height is influenced by genetic factors while 25% is by acquired factors [15]. Besides genetic factors, weight is influenced more by eating habits, nutrition level, and physical exercise. The mean values of BMI of Uygur male university students are higher than Han and Tibetan. And the mean values of BMI of Uygur and Tibetan female university students are higher than Han. The Han, Tibetan, and Uygur university students show no difference in obesity rate, and both male and female students show low obesity rates but high overweight rates. Overweight is the premonition of obesity, so it should be concerned by overweight and obese university students. The Han, Tibetan, and Uygur university students show different rates of underweight, normal weight, and overweight and present a high rate of underweight, normal weight, and overweight, respectively. The reasons may be as follows. (1) From the aspect of eating habits, traditional Tibetan foods include tsamba, ghee, milky tea, beef, mutton, and blood sausage. Traditional Uygur foods include crusty pancakes, hand meat, hand pilaf, stirred noodle, roast, roasted stuffed bun, and milky tea. Nutritionally, such traditional foods are rich in protein and fat, but high protein and high fat are recognized as hazards for obesity. Long-term intake of these foods will easily lead to fat accumulation and weight gain. The Han diet is mainly composed of cereals, vegetables, and fruits, so the intake of meat is lower than those of Tibetans and Uygurs. Most of the regular Uygur foods have high glycemic index, which is closely related to diabetes [16]. Fat accumulation for Uygur university students occurs mainly in abdomen, while abdomen obesity is more likely to cause diabetes [17]. (2) From the aspect of nutrition level, with the rapid economic development and the increasing living standard in China, the high-protein and high-fat foods that Tibetans and Uygurs prefer will more frequently enter regular households, and chronic intake of these foods will result in fat accumulation and weight gain. (3) From the aspect of physical exercise, Tibetans are nomad and mainly engaged in stockbreeding. They live in plateau with low temperature (7.5°C annually), so they have to ingest high-heat foods to resist cold. Uygurs are mainly devoted to agriculture and live in cold winter. Therefore, the Tibetan and Uygur diets correspond to their long-term living environment and labor consumption. However, little physical labor and little exercise result in little energy consumption by Tibetan and Uygur university students, so obesity will easily occur if they do not adjust eating habits. Though the results show the major proportion of Tibetan university students is normal weight, if they do not change their diet, obesity rate will gradually increase, which should alert Tibetan students. Most of the underweight university students are higher than the population's average weight, so malnutrition is caused by the failure of nutrition ingestion to meet the demands for growth and development. This is essentially different from malnutrition in small and short stature. Therefore, the physical condition of the underweight group should be improved by reasonable diet. The male university students of the three nations show no difference in the distribution of BMI, and the female students show the consistent distribution of BMI with the general comparison. Besides the above factors, this may be related to differences in aesthetics cultures, because Tibetan and Uygur females prefer a plump and trim figure, while Han females prefer a slender figure.

BMI increases with age for male students but decreases with age for female students. Female Han students show higher underweight and normal rates but lower overweight rate and obesity rate than male Han students. Female Tibetan students show higher normal weight rate but lower overweight rate and obesity rate than male Tibetan students. Male Uygur students show higher obesity rate than female Uygur students. This is mainly because female students, especially Han females, pay higher attention to figure than male students, so they purposely control diet.

Tibetan and Uygur university students have higher BMI than other minorities in South China, indicating that Tibetan and Uygur are typical minorities in Northwest China and show strong genetic differences from other minorities in South China. This also reflects the impact of regional and genetic factors on growth and development.

World Health Organization proposed that 60% of human health level is determined by behavior and living habits, 15% by genetic factors, 8% by medical and service conditions, and 7% by climate [18]. University students are at the early stage of adulthood and their physical functions are the optimal in lifecycle. Therefore, periodic physical examination should be taken for university students, so as to understand their physical conditions. Targeted health guidance should be performed to help them to form favorable eating and behavioral habits and to improve their life quality in the future.

## Figures and Tables

**Table 1:** Population distributions of Han, Tibetan, and Uygur university students.

Age	Han		Tibetan		Uygur	
	Male	Female	Male	Female	Male	Female
20	988	1019	140	161	50	110
21	955	779	120	143	54	100
22	1140	886	150	175	60	113
23	880	597	130	139	50	104
24	456	237	102	116	53	96

Total	4419	3518	642	734	267	523

**Table 2:** BMI distributions of Han, Tibetan, and Uygur university students.

Nationalities	Gender	*n*	BMI (kg/m^2^)			
			<18.5	18.5~23.9	24~27.9	≧28
Han	Male	4419	459 (10.39)^*⋇⋇*^	3340 (75.58)^*⋇⋇*^	493 (11.16)^*⋇⋇*^	127 (2.87)^*⋇⋇*^
	Female	3518	466 (13.25)^ΔΔ^	2771 (78.77)^Δ,Δ^	248 (7.05)^ΔΔ^	33 (0.94)

Total		7937	925 (11.65)**	6111 (76.99)**	741 (9.34)**	160 (2.02)

Tibetan	Male	642	55 (8.57)	496 (77.26)^*⋇*^	73 (11.37)^*⋇*^	18 (2.8)^*⋇⋇*^
	Female	734	71 (9.67)	604 (82.29)^ΔΔ^	52 (7.08)^ΔΔ^	7 (0.95)

Total		1376	126 (9.16)	1100 (79.94)**	125 (9.08)*	25 (1.82)

Uygur	Male	267	24 (8.99)	196 (73.41)	38 (14.23)	9 (3.37)^*⋇*^
	Female	523	69 (13.19)	388 (74.19)	61 (11.66)	5 (0.96)

Total		790	93 (11.77)	584 (73.92)	99 (12.53)	14 (1.77)

The comparison among Han, Tibetan, and Uygur University students: *P < 0.05, **P < 0.01. The female comparison among Han, Tibetan, and Uygur University students: ^Δ^ *P* < 0.05, ^ΔΔ^ *P* < 0.01. The comparison between males and females in the same nationality: ^⋇^ *P* < 0.05, ^⋇⋇^ *P* < 0.01.

**Table 3:** BMI of male and female university students at different ages.

	Gender	Amount	Age				
			20	21	22	23	24
BMI	Male	5328	21.20 ± 2.79	21.18 ± 2.74	21.36 ± 2.71	21.57 ± 2.76	21.74 ± 2.78
	Female	4775	21.00 ± 2.45	20.81 ± 2.23	20.72 ± 2.18	20.83 ± 2.40	20.61 ± 2.08

**Table 4:** Comparison of BMI averages between Tibetan and Uygur university students and other nations in South China.

Nationalities	Gender	Average BMI
Tibetan	male	21.35
	female	21
Uygur	male	21.81
	female	21.04
Ethnic minorities in Yunnan	male	20.31
	female	20.48
Yi nationality	male	20.39
	female	20.47
Sichuan Qiang	male	20.76
	female	19.31

**Table 5:** Comparison among BMI, gender and age in Tibetan, Uygur and Han university students ($\bar{x}\pm S$) Kg/m^2^.

Age	Han		Tibetan		Uygur	
	Male	Female	Male	Female	Male	Female
20	21.24 ± 2.70	20.82 ± 2.38	20.92 ± 2.29	21.26 ± 2.23	21.95 ± 3.89	21.05 ± 2.79
21	21.17 ± 2.78	20.71 ± 2.15	20.98 ± 2.59	21.02 ± 2.23	21.78 ± 2.32	21.15 ± 2.64
22	21.35 ± 2.72	20.67 ± 2.17	21.36 ± 2.55	20.90 ± 2.07	21.69 ± 2.93	20.90 ± 2.35
23	21.54 ± 2.76	20.80 ± 2.42	21.69 ± 2.79	20.67 ± 2.19	21.61 ± 2.68	21.17 ± 2.51
24	21.59 ± 2.73*	20.49 ± 2.10	22.26 ± 2.93	21.08 ± 2.02	22.61 ± 2.85	20.52 ± 2.00

Total	21.34 ± 2.76**	20.77 ± 2.29	21.35 ± 2.64*	21.00 ± 2.20*	21.81 ± 2.97	21.04 ± 2.53*

*P < 0.05, **P < 0.01.
